# High potency and water dispersible *Boswellia* extract reduces pain and inflammation in subjects with osteoarthritis of the knee: A randomized, double-blind, placebo-controlled trial

**DOI:** 10.1097/MD.0000000000050686

**Published:** 2026-09-18

**Authors:** Somashekara Nirvanashetty, Sanjib Kumar Panda

**Affiliations:** a Chief Scientific Officer, Olene Life Sciences Private Limited, Chennai, Tamil Nadu, India.

**Keywords:** *Boswellia* extract, *Boswellia serrata* extract, inflammation, knee osteoarthritis, OLNP-27, pain

## Abstract

**Background:**

*Boswellia serrata*, also known as Indian frankincense, is a medicinal plant that has been used since ancient times in the traditional Indian medicine system, Ayurveda. Historically, *Boswellia* extract has been used to treat pain associated with osteoarthritis, rheumatoid arthritis, and tendonitis.

**Methods:**

In this randomized, double-blind, placebo-controlled study, 60 subjects aged 40 to 75 years, with mild to moderate knee osteoarthritis received either 100 mg once daily of standardized natural *Boswellia* extract (OLNP-27) or placebo for 8-weeks. The subjects were monitored for outcome measures such as total Western Ontario and McMaster Universities Osteoarthritis Index (WOMAC) score, WOMAC subscales (pain, stiffness, and physical function) score and visual analogue score on day 7, 15, 30, and 60 post supplementation.

**Results:**

OLNP-27 significantly (*P* < .05) reduced the total WOMAC score, pain score, stiffness score, visual analogue score score and improved the physical function than the placebo. OLNP-27 also decreased the inflammatory marker high-sensitivity-CRP significantly compared to placebo group. The statistically significant reduction in pain and stiffness was observed as quickly as 7 days of supplementation with OLNP-27 indicating the quick onset of action. It also was found to be safe and well tolerated during this study.

**Conclusion:**

Based on the current study findings, it can be concluded that OLNP-27 is an effective and safe natural option for the management of symptoms associated with osteoarthritis of the knee.

## 1. Introduction

Knee osteoarthritis (OA) is a progressive and degenerative joint disease which is a result of progressive loss of articular cartilage in the knee joint. Knee OA may eventually lead to the complete disability and is one of the main causes of disability of elderly people across the globe. The symptoms include knee pain, knee stiffness and pain after prolonged sitting and resting which worsen over time. The purposes of conservative knee OA treatment are to alleviate pain, reduce the stiffness and to improve the function of the joint by pharmacological and non-pharmacological means. Wide variety of non steroidal anti-inflammatory drugs (NSAIDs) are used as 1st line therapy for the management of symptoms associated with knee OA followed by surgical methods, though no proven treatments are currently existing.

Associated side effects limit the usage of NSAIDs in long term. Long-term use of NSAIDs for OA is associated with enhanced risk of many side effects, including but not limited to gastrointestinal bleeding, hypertension, congestive heart failure, hyperkalemia and renal insufficiency. Although some of these disadvantages can be avoided by using selective cyclooxygenase 2 inhibitors, long-term use of these could lead to hepatotoxicity and chronic renal impairment and also found to cause cardiovascular side effects.^[1,2]^ Hence, natural alternatives for the treatment of Knee OA are considered as a safer option for long term usage.

*Boswellia serrata* is one of the ancient and most valued herbs in Ayurveda and has been used for centuries for pain relieving. The gum resin of *B serrata* contains monoterpenes, diterpenes, triterpenes, tetracyclic triterpene acids, and pentacyclic triterpene acids called boswellic acids (BAs) which are responsible for health benefits.^[3]^ 3-Acetyl-11-keto-beta-boswellic acid (AKBA) is the most potent active compound among the boswellic acids and has shown significant pharmacological activity in the treatment of inflammatory diseases such as OA, rheumatoid arthritis, chronic bronchitis, asthma and chronic inflammatory bowel diseases.^[4]^

The main mechanism of anti-inflammatory activity includes inhibition of 5-lipoxygenase (5-LOX), thereby reducing the production leukotrienes or activation of inflammatory mediators such as matrix metalloproteinase-9, matrix metalloproteinase-13, cyclooxygenase-2, and nitric oxide as well as analgesic and anti-arthritic effects.^[5]^ Inhibition of pro-inflammatory enzyme, 5-LOX inhibits the production of leukotrienes responsible for the inflammation. As potential anti-inflammatory treatment for OA, the efficacy of *Boswellia* extracts has been proven in several animal and human clinical studies.^[4,6]^ However, its therapeutic activity is limited due to its poor aqueous solubility, poor oral bioavailability and higher phase-1 metabolism.^[7,8]^

Olene Life Sciences Private Limited thus successfully developed a natural and water dispersible *Boswellia* extract, OLNP-27 standardized to >40% AKBA by high performance liquid chromatography. Water dispersibility enhances the absorption of AKBA and hence its bioavailability and bio-efficacy. The objective of this study was to evaluate the efficacy and safety of OLNP-27 versus placebo in reducing symptoms of knee OA in subjects with mild to moderate knee OA.

## 2. Materials and methods

### 2.1. Study design

**Figure 1.**
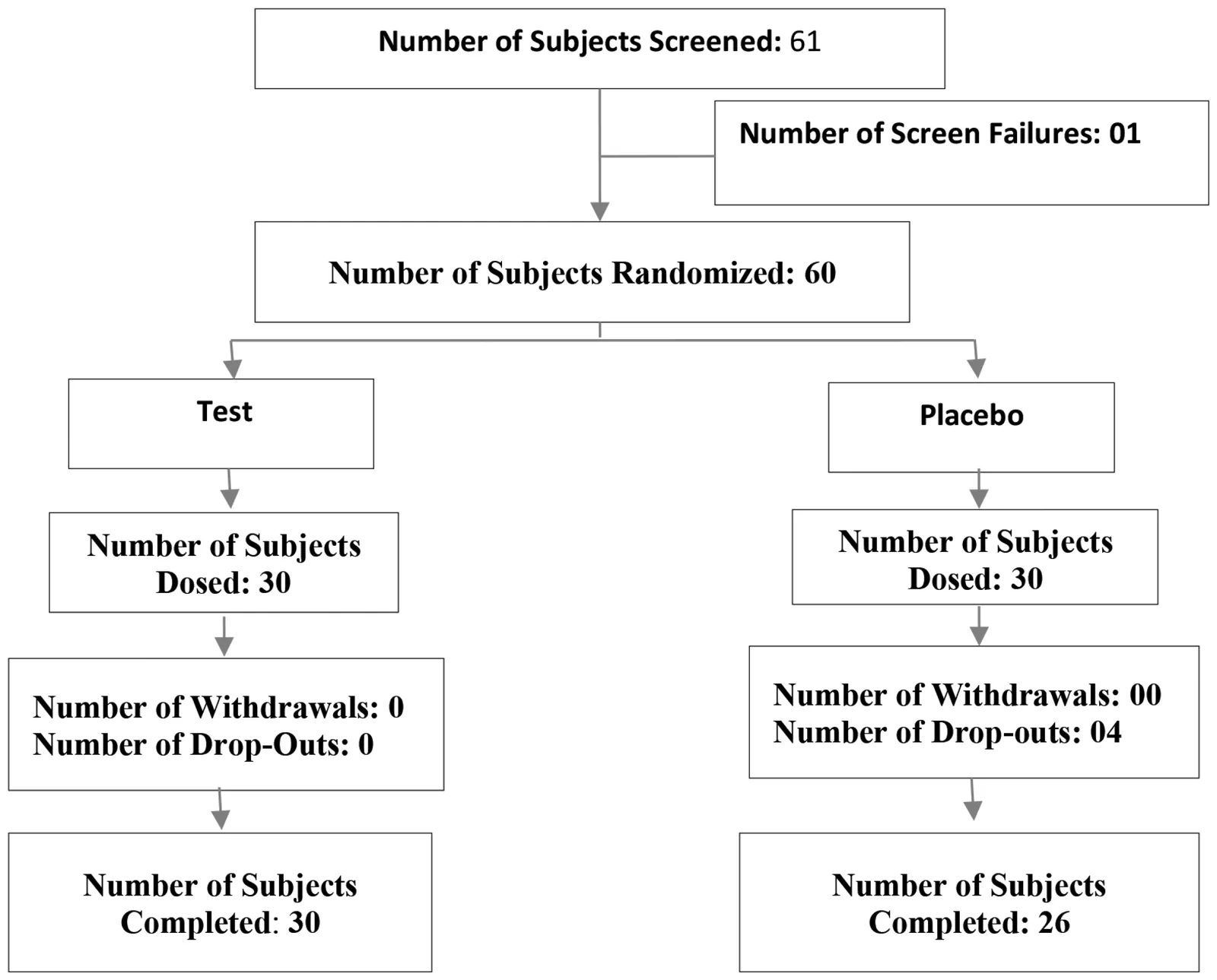
Study flow chart.

This study was a randomized, double-blind, placebo-controlled, parallel-group design over an 8-week (60 days) period to assess the safety and efficacy of OLNP-27 in alleviating symptoms associated with knee OA. Participants were selected based on predefined inclusion and exclusion criteria. The study was performed in Chennai Meenakshi Multispecialty Hospital, Chennai, India. After obtaining informed consent, eligible subjects were randomly assigned in a 1:1 ratio to either the OLNP-27 group (test group) or the placebo group. The study duration of 60 days included 6 key assessment points: screening visit (day −7), baseline visit (visit 1, day 0), and follow-up visits (visit 2, day 7; visit 3, day 15; and visit 4, day 30), and the final visit (visit 5, day 60). Efficacy and safety parameters, comprising the total Western Ontario and McMaster Universities Osteoarthritis Index (WOMAC) score, WOMAC subscales (pain, stiffness, and physical function), visual analogue scale (VAS) pain score, serum high-sensitivity C-reactive protein (hs-CRP) levels, and adverse events (AEs), were evaluated throughout the study duration. Furthermore, physical examinations and vital signs were assessed at each visit. Data collection was carried out utilizing paper-based case report forms. The flow of study participants is depicted in Figure 1.

### 2.2. Study population and sample size

**Figure 2.**
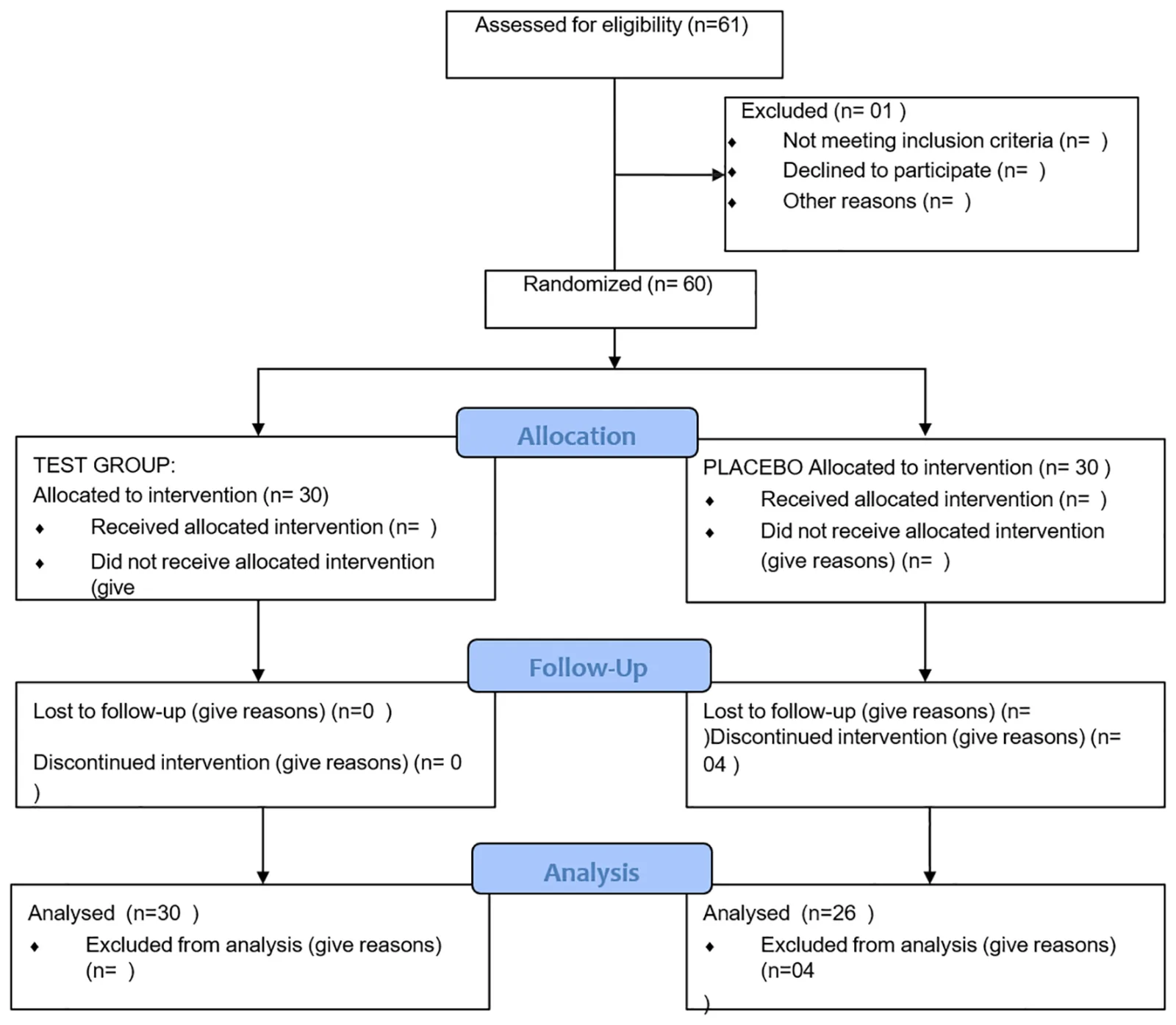
CONSORT 2010 flow diagram. CONSORT = Consolidated Standards of Reporting Trials.

This study recruited individuals diagnosed with unilateral or bilateral OA of the knee. All eligible participants willingly consented to participate in the study. With an anticipated dropout rate of 10%, a total of 60 subjects were recruited to ensure at least 56 participants completed the study. The Consolidated Standards of Reporting Trials flow diagram (Fig. 2) demonstrates how participants were enrolled, allocated to the study groups, monitored during the study period, and included in the final analysis. The diagram also provides transparency regarding participant exclusions and withdrawals, thereby ensuring the integrity of the study’s conduct and analysis.^[9]^

### 2.3. Inclusion and exclusion criteria

Male and female individuals aged between 40 and 75 years, diagnosed with unilateral or bilateral knee OA as per the criteria established by the American College of Rheumatology (ACR), with symptoms persisting for more than 3 months and radiographic confirmation of Kellgren-Lawrence grade 2 or 3, were included in this study. Additionally, individuals were required to have a VAS pain score ranging from 40 to 70 mm during the most painful knee movement. Female participants of reproductive age were mandated to utilize an approved form of contraception. Additionally, female participants who were incapable of conception due to either 1 year of amenorrhea or a documented history of hysterectomy and/or bilateral oophorectomy were also considered eligible for inclusion.

The exclusion criteria included subjects with a history of inflammatory arthropathy, severe rheumatoid arthritis, or OA, as well as those scheduled for surgery within 3 months or who had experienced a recent knee injury within the past 4 months from the screening date. Additionally, exclusion criteria included abnormal liver/kidney function, renal insufficiency, endocrine disorders, or conditions hindering study outcomes. Participants were also excluded if they had used corticosteroids, indomethacin, glucosamine, or other supplements within 3 to 6 months prior, or had significant hematological, renal, pulmonary, gastrointestinal, cardiovascular, hepatic, neurological/psychiatric disorders, or malignancies. Moreover, participants with a history of excessive alcohol intake (>2 standard drinks/d), substance abuse, or women who were pregnant, breastfeeding, or planning pregnancy were also excluded.

### 2.4. Interventions

Eligible participants were randomly allocated in a 1:1 ratio to 1 of 2 treatment groups: the test group and the placebo group. Participants in the test group were administered OLNP-27 capsules (100 mg), while those in the placebo group received placebo capsules (100 mg). All participants were instructed to consume 1 capsule daily for 60 days. Paracetamol (2000 mg/day) was administered as rescue medication under the investigator’s guidance. The use of rescue medication was documented in the source documents, subject diary cards, and case report forms. Subjects were instructed to take the rescue medication 24 hours before their site visit.

### 2.5. Outcome measures

#### 2.5.1. Primary efficacy variables

The primary efficacy variable in this study focused on assessing the mean change in total WOMAC score from baseline to the end of the study. The WOMAC index utilizes Likert scales that allow subjects to self-assess the status of their OA condition. It evaluates 3 subscales: pain, stiffness, and physical function associated with OA, along with a total score ranging from 0 (indicating optimal status) to 96 (reflecting the most severe impairment). In cases where participants had OA in both knees, they were instructed to select the more painful knee as the primary knee for assessment. Higher scores on the WOMAC index indicate greater pain and stiffness, as well as decreased physical function. This assessment was conducted during each visit throughout the study period.

#### 2.5.2. Secondary efficacy variables

The secondary efficacy variables included the mean change from baseline in the VAS pain score, WOMAC subscale scores (pain, stiffness, and physical function), and the levels of serum hs-CRP as an inflammatory marker. The VAS is a commonly used tool for pain assessment, represented by a horizontal line ranging from 0 to 100, with word descriptors at each end. Pain levels were categorized as follows: 0 to 4 mm indicating no pain; 5 to 44 mm indicating mild pain; 45 to 74 mm indicating moderate pain; and 75 to 100 mm indicating severe pain. Assessments of VAS score and WOMAC subscale scores were conducted at screening and during every follow-up visit (baseline, day 7, day 15, day 30, and day 60), while hs-CRP levels were evaluated at baseline and during visit 5 (day 60).

#### 2.5.3. Tolerability and safety assessment

Tolerability was evaluated by documenting the occurrence, duration, and severity of all AEs, as well as instances of patient withdrawal attributable to AEs. Additionally, safety was comprehensively assessed through monitoring vital signs and conducting physical examinations.

### 2.6. Ethical considerations

The study protocol was approved by the Institutional Ethics Committee of Chennai Meenakshi Multispecialty Hospital, Chennai, India. The study adhered to the principles outlined in the Declaration of Helsinki, the ICMR ethical guidelines for biomedical research, and the International Conference on Harmonization of Good Clinical Practices. The study was prospectively registered with the Clinical Trials Registry-India (CTRI No. CTRI/2022/01/039859). All study objectives and procedures were clearly explained to participants, and informed consent was obtained from each participant prior to enrollment.

### 2.7. Statistical analyses

An intention-to-treat population analysis was used to assess efficacy. Descriptive statistics were employed to summarize demographic and baseline characteristics. Data were presented as the arithmetic mean, standard deviation, and percentages. Both the primary and secondary outcomes were analyzed using a paired *t* test for within-group comparisons and an unpaired *t* test for between-group comparisons. Data analysis was performed using the SPSS software. A *P* value ≤.05 was considered statistically significant.

## 3. Results

### 3.1. Baseline characteristics

A total of 61 subjects were screened, of which 1 subject did not fulfill the eligibility criteria and was considered a screen failure. Out of the total 60 subjects enrolled, 30 subjects were allocated to the OLNP-27 group and 30 subjects were allocated to the placebo group. Fifty-six subjects completed the study as per protocol requirements: 30 subjects in the OLNP-27 group and 26 in the placebo group. The disposition of subjects is shown in Figure 1. There were no significant differences at baseline between both groups regarding age, height, weight, body mass index, VAS, total WOMAC, and WOMAC subscale score. The comparative baseline characteristics and demographic variables of the subjects are shown in Table 1.

**Table 1:** Demographics and baseline characteristics (ITT population).

	OLNP-27	Placebo
	(N = 30)	(N = 30)
Age (yr)		
Mean (standard deviation)	53.27 ± 8.12	51.93 ± 8.12
Median	51.50	50.5
Range	40–67	40–76
Gender, N (%)		
Male	10 (33.33%)	8 (26.67%)
Female	20 (66.67%)	22 (73.33%)
Weight (kg)		
Mean (standard deviation)	65.79 ± 11.17	66.16 ± 10.09
Median	67.85	64.65
Range	42.5–84	51.4–88.2
Height (cm)		
Mean (standard deviation)	158.99 ± 7.74	156.67 ± 7.27
Median	157.50	155
Range	146–172	144–173
BMI (kg/m^2^)		
Mean (standard deviation)	25.88 ± 3.08	26.81 ± 2.33
Median	26.05	26.95
Range	19.1–29.9	21–30.5

BMI = body mass index, ITT = intention-to-treat, OLNP-27.

### 3.2. Primary efficacy outcome measure

As shown in Table 2, a significant reduction in total WOMAC score was observed in the OLNP-27 group compared to the placebo group. In within-group analysis, the difference was found to be statistically significant (*P* < .05) at all post-baseline visits in the OLNP-27 group. Supplementation of OLNP-27 for 60 days led to a 63.41% reduction in total WOMAC score versus 17.26% in the placebo group. The significant reduction in total WOMAC score was seen as early as day 7 of OLNP-27 supplementation.

**Table 2:** Mean change from baseline in total WOMAC (ITT population).

	Mean change from baseline in total WOMAC			
	Placebo N = 30		OLNP-27 N = 30	
Visits	Mean (SD)	Change from baseline	Mean (SD)	Change from baseline
Baseline (day 0)	5.93 ± 0.35		5.91 ± 0.41	
Intention-to-treat (day 7)	4.74 ± 0.57*,†	1.19 ± 0.56	5.59 ± 0.42	0.32 ± 0.25
Visit 3 (day 15)	3.96 ± 0.52*,†	1.97 ± 0.54	5.26 ± 0.46	0.65 ± 0.35*
Visit 4 (day 30)	2.77 ± 0.50*,†	3.16 ± 0.60	5.08 ± 0.47	0.83 ± 0.45*
Visit 5 (day 60)	2.17 ± 0.79*,†	3.76 ± 0.77	4.89 ± 0.53	1.02 ± 0.49*

ITT = intention-to-treat, OLNP-27, SD = standard deviation, WOMAC = Western Ontario and McMaster Universities Osteoarthritis Index.

* Statistically significant (*P* < .05) within group.

† Statistically significant (*P* < .05) between group. Within group analysis by paired *t* test and between group analyses by unpaired *t* test.

### 3.3. Secondary efficacy outcome measure

As shown in Tables 3–6, there was a significant (*P* < .05) reduction in VAS, WOMAC pain, WOMAC stiffness, and physical function scores in the OLNP-27 group compared to the placebo group. Within-group analysis showed that supplementation of OLNP-27 significantly (*P* < .05) reduced the VAS, WOMAC pain, WOMAC stiffness, and physical function scores in all post-supplementation visits.

**Table 3:** Mean change from baseline in VAS (ITT population).

	Mean change from baseline in VAS			
Visits	OLNP-27 N = 30		Placebo N = 30	
	Mean (SD)	Change from baseline	Mean (SD)	Change from baseline
Baseline (day 0)	67.95 ± 2.23		68.07 ± 2.36	
Visit 2 (day 7)	59.89 ± 5.15	8.06 ± 5.42*,†	66.28 ± 2.79	1.79 ± 1.81*
Visit 3 (day 15)	52.85 ± 4.16	15.10 ± 4.61*,†	64.23 ± 3.50	3.84 ± 3.19*
Visit 4 (day 30)	31.92 ± 9.23	36.03 ± 9.80*,†	62.09 ± 4.16	5.98 ± 4.21*
Visit 5 (day 60)	26.39 ± 10.63	41.56 ± 11.06*,†	59.52 ± 4.77	8.55 ± 5.13*

ITT = intention-to-treat, OLNP-27, SD = standard deviation, VAS = visual analogue score.

* Statistically significant (*P* < .05) within group.

† Statistically significant (*P* < .05) between group. Within group analysis by paired *t* test and between group analysis by unpaired *t* test.

**Table 4:** Mean change from baseline in WOMAC pain score (ITT population).

	Mean change from baseline in WOMAC pain score			
Visits	OLNP-27 N = 30		Placebo N = 30	
	Mean (SD)	Change from baseline	Mean (SD)	Change from baseline
Baseline (day 0)	1.95 ± 0.25		2.03 ± 0.21	
Visit 2 (day 7)	1.65 ± 0.22	0.29 ± 0.28*,†	1.93 ± 0.21	0.10 ± 0.13*
Visit 3 (day 15)	1.41 ± 0.20	0.53 ± 0.27*,†	1.88 ± 0.18	0.15 ± 0.15*
Visit 4 (day 30)	0.94 ± 0.20	1.01 ± 0.29*,†	1.79 ± 0.18	0.24 ± 0.18*
Visit 5 (day 60)	0.73 ± 0.29	1.22 ± 0.29*,†	1.77 ± 0.19	0.27 ± 0.18*

ITT = intention-to-treat, OLNP-27, SD = standard deviation, WOMAC = Western Ontario and McMaster Universities Osteoarthritis Index.

* Statistically significant (*P* < .05) within group.

† Statistically significant (*P* < .05) between group. Within group analysis by paired *t* test and between group analysis by unpaired *t* test.

**Table 5:** Mean change from baseline in WOMAC stiffness score (ITT population).

	Mean change from baseline in WOMAC stiffness score			
	OLNP-27 N = 30		Placebo N = 30	
Visits	Mean (SD)	Change from baseline	Mean (SD)	Change from baseline
Baseline (day 0)	1.97 ± 0.18		1.88 ± 0.25	
Visit 2 (day 7)	1.43 ± 0.34	0.53 ± 0.35*,†	1.78 ± 0.28	0.10 ± 0.20
Visit 3 (day 15)	1.15 ± 0.30	0.82 ± 0.33*,†	1.63 ± 0.31	0.26 ± 0.28*
Visit 4 (day 30)	0.80 ± 0.25	1.17 ± 0.33*,†	1.60 ± 0.31	0.28 ± 0.31*
Visit 5 (day 60)	0.62 ± 0.34	1.35 ± 0.37*,†	1.52 ± 0.31	0.37 ± 0.32*

ITT = intention-to-treat, OLNP-27, SD = standard deviation, WOMAC = Western Ontario and McMaster Universities Osteoarthritis Index.

* Statistically significant (*P* < .05) within group.

† Statistically significant (*P* < .05) between group. Within group analysis by paired *t* test and between group analysis by unpaired *t* test.

**Table 6:** Mean change from baseline in WOMAC physical function (ITT population).

	Mean change from baseline in WOMAC physical function			
Visits	OLNP-27 N = 30		Placebo N = 30	
	Mean (SD)	Change from baseline	Mean (SD)	Change from baseline
Baseline (day 0)	2.02 ± 0.10		1.99 ± 0.13	
Visit 2 (day 7)	1.66 ± 0.20	0.36 ± 0.23*,†	1.88 ± 0.13	0.12 ± 0.11
Visit 3 (day 15)	1.40 ± 0.13	0.62 ± 0.18*,†	1.76 ± 0.16	0.24 ± 0.16*
Visit 4 (day 30)	1.03 ± 0.19	0.99 ± 0.24*,†	1.68 ± 0.19	0.31 ± 0.20*
Visit 5 (day 60)	0.83 ± 0.25	1.19 ± 0.28*,†	1.61 ± 0.21	0.39 ± 0.22*

ITT = intention-to-treat, OLNP-27, SD = standard deviation, WOMAC = Western Ontario and McMaster Universities Osteoarthritis Index.

* Statistically significant (*P* < .05) within group.

† Statistically significant (*P* < .05) between group. Within group analysis by paired *t* test and between group analysis by unpaired *t* test.

Supplementation of OLNP-27 for 60 days led to a 62.56%, 68.53%, 58.91%, and 61.16% reduction in WOMAC pain score, WOMAC stiffness score, WOMAC physical function score, and VAS score respectively, whereas the percentage reduction in the placebo group was 12.81%, 19.15%, 19.09%, and 12.56% for WOMAC pain score, WOMAC stiffness score, WOMAC physical function score, and VAS score respectively.

Significant reductions in pain, stiffness, and physical function scores were observed as early as on day 7 in the OLNP-27 group compared to baseline and to the placebo group, which indicates the quick onset of action of OLNP-27.

Supplementation of OLNP-27 for 60 days resulted in a 78.9% (*P* < .05, baseline: 3.98 ± 3.01 mg/L vs. day 60: 0.84 ± 0.96 mg/L) reduction in serum hs-CRP as compared to 10.4% (baseline: 4.25 ± 6.55 mg/L vs. day 60: 3.81 ± 5.45 mg/L) in the placebo group (Table 7).

**Table 7:** Mean change from baseline in hs-CRP (ITT population).

	Mean change from baseline in hs-CRP (mg/L)			
Visits	OLNP-27 N = 30		Placebo N = 30	
	Mean (SD)	Change from baseline	Mean (SD)	Change from baseline
Baseline (day 0)	3.98 ± 3.01		4.25 ± 6.55	
Visit 5 (day 60)	0.84 ± 0.96	3.14 ± 2.62*,†	3.81 ± 5.45	0.44 ± 1.94

hs-CRP = high-sensitivity C-reactive protein, ITT = intention-to-treat, OLNP-27, SD = standard deviation.

* Statistically significant (*P* < .05) within group.

† Statistically significant (*P* < .05) between group. Within group analysis by paired *t* test and between group analysis by unpaired *t* test.

### 3.4. Rescue medication usage

Rescue medication was provided by the study physician after investigation, and the use of rescue medication was recorded. Thirty percent of subjects (9 subjects) in the placebo group used rescue medication, whereas only 10% of the subjects (3 subjects) used rescue medication in the OLNP-27 group. The total number of rescue medications taken by the subjects in the OLNP-27 group was only 11 tablets of 500 mg paracetamol, whereas in the placebo group, it was 75 tablets of 500 mg paracetamol. There was minimal use of rescue medication in the OLNP-27 group than the placebo group.

### 3.5. Adverse events

The overall incidence of AEs was 3.33% (1/30 subjects) and 13.33% (4/30 subjects) in the OLNP-27 and placebo groups, respectively. There was no statistically significant difference in the incidence of AEs between the OLNP-27 and placebo groups. All AEs were mild to moderate in severity in both groups, and no clinically relevant AEs were identified. The AE in the OLNP-27 group was diarrhea 1 (3.33%), and in the placebo group were cold and cough 1 (3.33%), diarrhea 1 (3.33%), cold 1 (3.33%), and stomach pain 1 (3.33%). AEs were classified by System Organ Class (Body System)/Preferred Term across groups.

### 3.6. Dropout

Four subjects from the placebo group were lost to follow-up.

## 4. Discussion

*B serrata* extract has been found to be a promising candidate for alleviating arthritic conditions. The main active ingredients of *Boswellia* extract are BAs, which have been shown to contribute to most of the pharmacological activity. Among these, AKBA has been found to be the most potent biologically active boswellic acid and has been extensively researched for its positive health effects. AKBA showed strong analgesic, anti- inflammatory, and anti-arthritic activity by noncompetitive inhibition of 5-LOX, leukocyte esterase, serine protease-cathepsin G, and microsomal prostaglandin E synthase^[10–14]^ AKBA has been found to reduce cartilage degradation by lowering glycosaminoglycan breakdown and enhancing glycosaminoglycan synthesis^.[15,16]^ Despite its broad therapeutic potential, previous research findings demonstrated poor oral bioavailability of AKBA, and the observed serum concentration was much lower than the IC50 value (3.0 µmol) for 5-LOX inhibition observed in vitro^.[17,18]^ A variety of *Boswellia* products are widely available on the market. Most of them are standardized at 1%–30% AKBA and have been shown to improve symptoms of OA. Despite their AKBA content, these products have significant drawbacks, including a strong resinous taste and smell, poor water dispersibility, and poor oral bioavailability.

**Figure 3.**
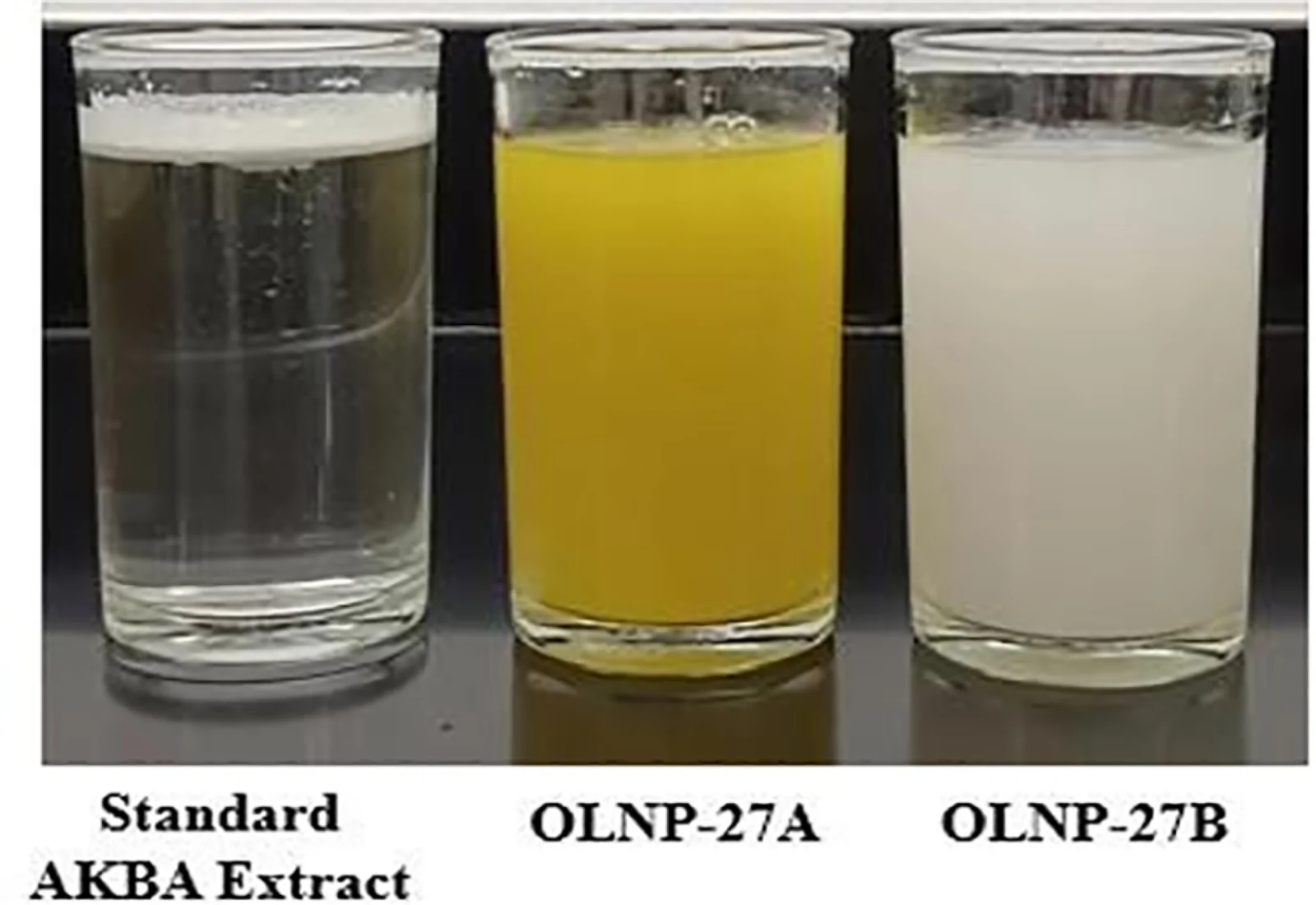
Dispersion of standard AKBA and OLNP-27. AKBA = 3-acetyl-11-keto-beta-boswellic acid, OLNP-27.

OLNP-27 is a natural, water-dispersible, highly concentrated *Boswellia* extract standardized to >40% AKBA by high performance liquid chromatography. It’s a highly absorbable formulation made using patent-pending, clinically verified Self-D technology. The dispersion analysis (Fig. 3) revealed that *B serrata*, OLNP-27, exhibited enhanced dispersibility in aqueous media. Enhanced dispersibility of hydrophobic molecules, which otherwise form clusters, increases intestinal absorption through increased surface area, ultimately leading to improved bioavailability.^[8]^ OLNP-27 addresses all the shortcomings of existing standard *Boswellia* extracts, including lower AKBA concentrations (1%–3%), non- dispersibility, poor bioavailability, a strong resinous smell, limited applications (only for capsules and tablets), and high levels of contaminants (residual solvents, heavy metals, aflatoxins, and pesticides).^[19]^ Additionally, OLNP-27 is palate-friendly because of its neutral taste and smell, requiring no or limited flavoring or masking agents as compared to regular standard extract, which has a strong resinous smell. Therefore, it is suitable for a variety of formulations, both in conventional and novel formats.

In this randomized, double-blind, placebo-controlled study, in comparison with placebo, supplementation of OLNP-27 for 8-weeks, at a dose of 100 mg once daily, significantly reduced the symptoms associated with knee OA, including pain, joint stiffness and improved physical function in adult participants. Supplementation of OLNP-27 for 60 days lead to 62.56%, 68.53%, 58.91%, and 61.16% reduction in WOMAC pain score, WOMAC stiffness score, WOMAC physical function score, and VAS score respectively. These results are well corroborated with findings from earlier human clinical studies with standardized *Boswellia* extracts.^[20,21]^ In this study, the statistically significant reduction in total pain, joint stiffness and improvement in physical function was seen as early as day 7 of OLNP-27 supplementation. This quick onset of action may be attributed to the higher levels of AKBA in OLNP-27 and its water dispersibility which leads to the enhanced bioavailability. Increased water dispersion of hydrophobic phytochemicals is expected to enhance the bioavailability and hence the bio-efficacy.^[8]^

As per the human clinical study, OA patients with elevated hs-CRP levels have increased cytokine levels and inflammatory changes.^[22]^ Elevated levels of hs-CRP have been correlated with the severity of OA symptoms such as pain, stiffness, and its progression. In this current study, the levels of serum hs-CRP were reduced by 78.9%, and this decrease in hs-CRP was very well correlated with the percentage decrease in pain and stiffness score.

## 5. Conclusion

The current study concludes that supplementation of OLNP-27 at a dose of 100 mg once daily was effective in significantly reducing symptoms associated with OA in subjects with mild to moderate knee OA. It was also found to be safe and well tolerated during the study. OLNP-27 showed statistically significant (*P* < .05) and clinically meaningful improvement in pain, stiffness, and physical function score. The present study results confirmed that OLNP-27 can be an effective and safe alternative therapy for the management of mild to moderate symptoms associated with knee OA. However, this study was carried out in a smaller population, and hence a larger clinical trial involving a larger group and longer duration with varying doses of OLNP-27 is thus warranted.

## Acknowledgments

The authors are grateful to Dr R. Vani Raj for managing the clinical studies and providing valuable input.

## Author contributions

**Conceptualization:** Somashekara Nirvanashetty.

**Methodology:** Sanjib Kumar Panda.

**Writing – original draft:** Sanjib Kumar Panda.
